# Naringenin derivatives as glucosamine-6-phosphate synthase inhibitors: synthesis, antioxidants, antimicrobial, preservative efficacy, molecular docking and in silico ADMET analysis

**DOI:** 10.1186/s13065-020-00693-3

**Published:** 2020-06-19

**Authors:** Amit Lather, Sunil Sharma, Anurag Khatkar

**Affiliations:** 1 Department of Pharmaceutical Sciences, Maharshi Dayanand University, Rohtak, Haryana India; 2 Department of Pharmaceutical Sciences, G.J.U.S.&T., Hisar, India; 3 Laboratory for Preservation Technology and Enzyme Inhibition Studies, Department of Pharmaceutical Sciences, Faculty of Pharmaceutical Sciences, Maharshi Dayanand University, Rohtak, Haryana India

**Keywords:** G-6-P synthase, Naringenin derivatives, DPPH, Preservative efficacy

## Abstract

**Background:**

Preservatives have to be added in food, pharmaceuticals and cosmetics products to maintain their shelf life. However, the existing chemical based preservatives have been associated with severe side effects that compel the researchers to find better safe preservatives based on natural products. G-6-P synthase is an important enzyme for bacterial and fungal cell wall synthesis and offers as a potential target to find better G-6-P synthase inhibitors based antimicrobial compounds. Naringenin, a flavanone, has been reported for a wide range of pharmacological activities including antimicrobial activity, which makes it a potential candidate to be explored as novel G-6-P synthase inhibitor.

**Results:**

The synthesis of naringenin derivatives with potent G-6-P synthase inhibitor having remarkable antioxidant, antimicrobial and preservative efficacy was performed. Among the synthesized compounds, the compound **1** possessed good antioxidant activity (IC_50_ value, 6.864 ± 0.020 µM) as compared to standard ascorbic acid (IC_50_ value, 8.110 ± 0.069 µM). The antimicrobial activity of synthesized compounds revealed compound **1** as the most potent compound (pMIC 1.79, 1.79, 1.49, 1.49, 1.49 and 1.49 μM/mL for *P. mirabilis, P. aeruginosa, S. aureus*, *E. coli, C. albicans* and *A. niger* respectively) as compared to standard drugs taken. The compound **2** showed comparable activity against *P. mirabilis* (pMIC 1.14 μM/mL)*, C. albicans* (pMIC 1.14 μM/mL) while the compound **3** also showed comparable activity against *C. albicans* (pMIC 1.16 μM/mL) as well *A. niger* (pMIC 1.46 μM/mL), likewise the compound **4** showed comparable activity against *P. mirabilis* (pMIC 1.18 μM/mL) as compared to the standard drugs streptomycin (pMIC 1.06, 1.36, 1.06 and 1.96 μM/mL for *P. mirabilis, P. aeruginosa, S. aureus* and *E. coli* respectively), ciprofloxacin (pMIC 1.12, 1.42, 1.12 and 1.42 μM/mL for *P. mirabilis, P. aeruginosa, S. aureus* and *E. coli* respectively), ampicillin (pMIC 1.14, 0.84, 0.84 and 1.74 μM/mL for *P. mirabilis, P. aeruginosa, S. aureus* and *E. coli* respectively) and fluconazole (pMIC 1.08 and 1.38 μM/mL for *C. albicans* and *A. niger* respectively). The molecular docking with the target G-6-P synthase pdb id 1moq resulted with an better dock score for compound **1** (− 7.42) as compared to standard antimicrobial drugs, ciprofloxacin (− 5.185), ampicillin (− 5.065) and fluconazole (− 5.129) that supported the wet lab results. The preservative efficacy test for compound **1** in White Lotion USP showed the log CFU/mL value within the prescribed limit and results were comparable to standard sodium benzoate, ethyl paraben and propyl paraben as per USP standard protocol.

**Conclusions:**

The synthesized naringenin derivatives exhibited significant G-6-P synthase inhibitory potential with good selectivity towards the selected target G-6-P synthase. Compound **1**, bearing nitro group showed good antioxidant, antimicrobial and preservative efficacy compared with the standard drugs taken. The mechanistic insight about the compounds within the active site was completed by molecular docking that supported the results for novel synthesized G-6-P synthase inhibitors.
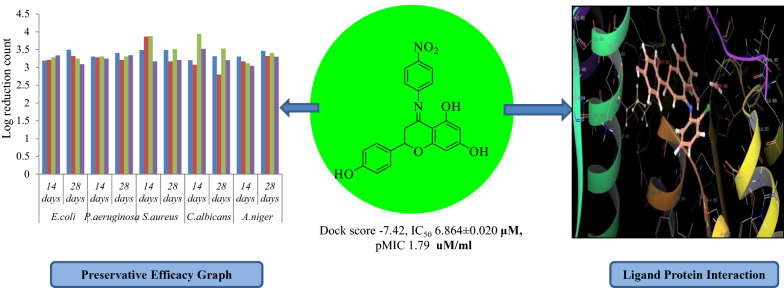

## Introduction

The use of packaged foods containing various additive’s viz. artificial sweeteners, colorants, stabilizers, preservatives etc. has greatly increased in recent years. As per recent data available it is estimated that 75% of the contemporary diet is packaged food and on an average every person consumes 3.6 to 4.5 kg of food additives per year [1].

Among other additives the preservative such as sodium benzoate, ethyl paraben, propyl paraben, butylated hydroxytoluene (BHT), butylated hydroxyanisole (BHA), etc. plays a vital role to maintain the shelf life of various food, pharmaceuticals and cosmetic products [2–4]. However, the existing chemical preservatives have been associated with serious side effects viz. estrogenic effect, breast cancer, malignant melanoma, contact eczema, endocrine disruption, etc. [5–12]. Hence, there is an urgent need for the discovery of novel and safer preservatives for use in food, pharmaceuticals and cosmetic products.

G-6-P synthase is a complex enzyme involved in the formation of UDP-N-acetyl glucosamine and catalyzes the initial step in hexosamine biosynthesis. One of these catalyzed products, N-acetyl glucosamine, is an important part of the peptidoglycan layer of bacterial and fungal cell wall. Hence, G-6-P synthase may act as potential target for discovery of novel antimicrobial compounds which could be evaluated for their preservative efficacy to find better and safe preservatives [13, 14].

**Fig. 1:**
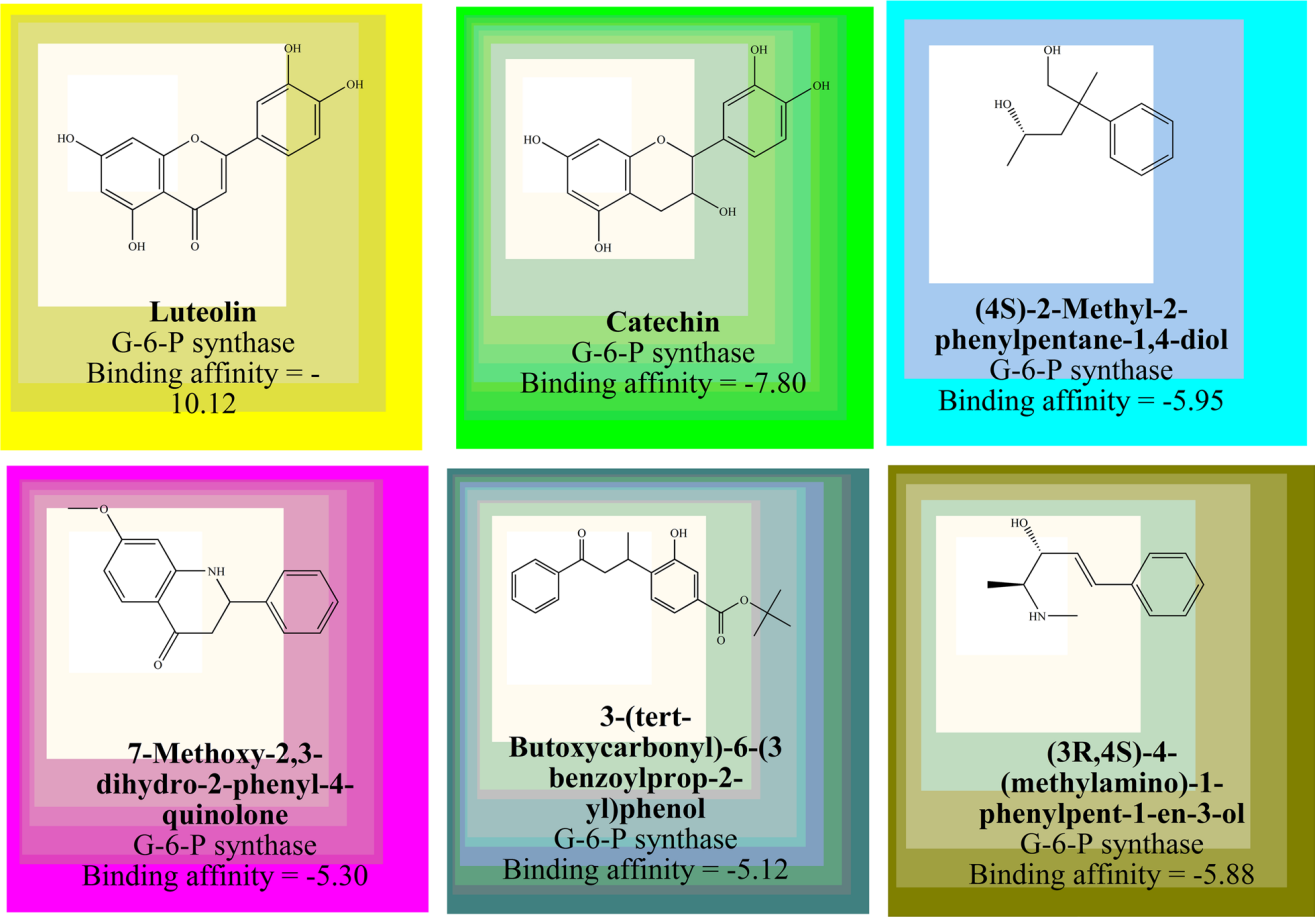
G-6-P synthase inhibitory profile of flavonoids and their derivatives cited in the recent literature

The complex 3-D crystal structure of G-6-P synthase can be utilized for molecular docking to explore the structural requirements for the pharmacophore complex. Flavonoids such as luteolin, catechin, (4S)-2-Methyl-2-phenylpentane-1,4-diol, 7-Methoxy-2,3-dihydro-2-phenyl-4 quinolone, 3-(tert-Butoxycarbonyl)-6-(3 benzoylprop-2-yl)phenol and (3R,4S)-4-(methylamino)-1-phenylpent-1-en-3-ol also have been explored for G-6-P synthase inhibition [15–18]. Some flavonoids along with their G-6-P synthase inhibitory dock score have been shown in Fig. 1.

**Fig. 2:**
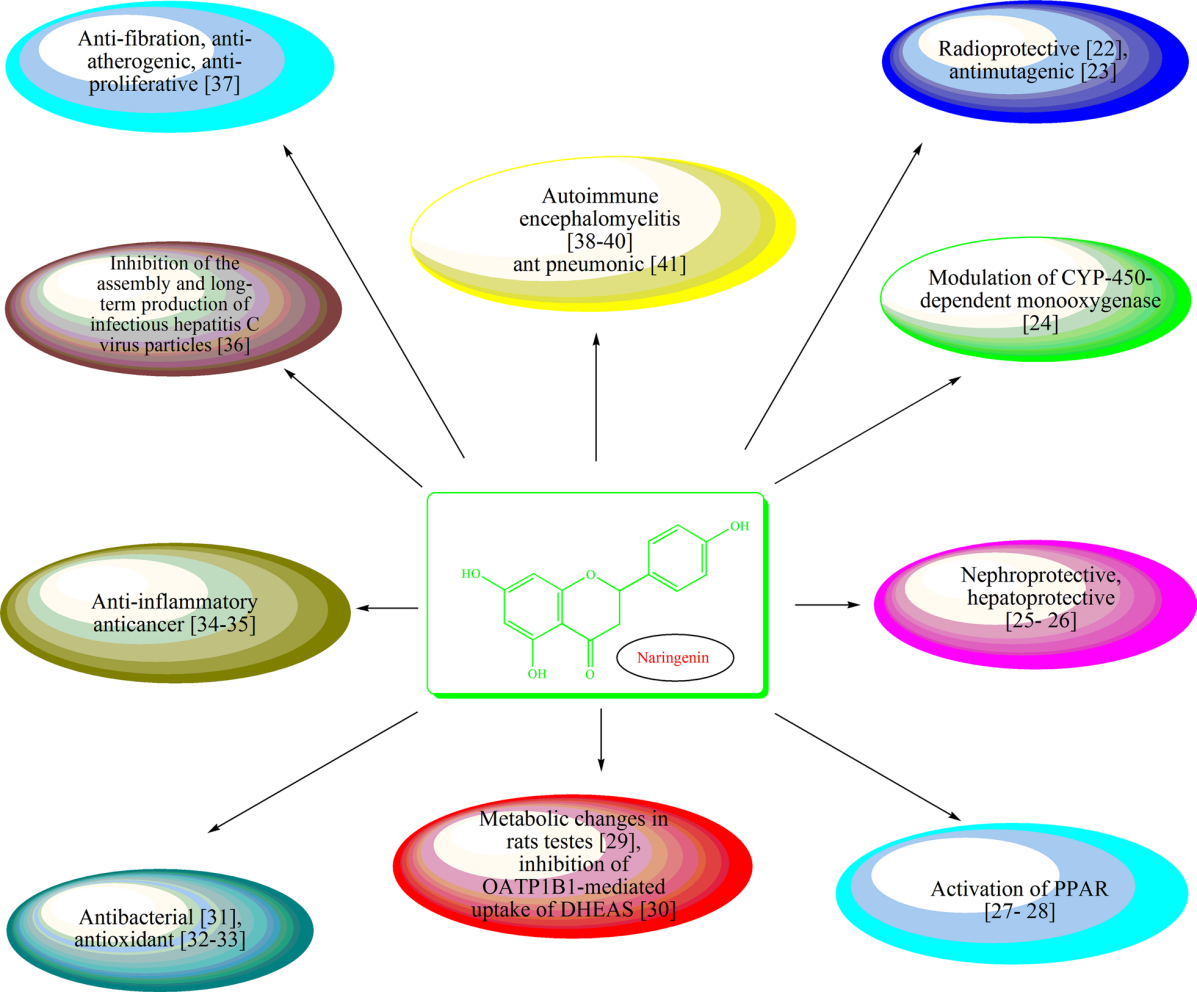
Pharmacological potential of Naringenin

Naringenin is a naturally occurring bioflavonoid present in various fruits, vegetables and honey which is used as a dietary supplement due to its low toxicity [19–21]. Naringenin has been reported for its diverse pharmacological profile including its antibacterial property as shown in Fig. 2 [22–41].

Further, naringenin could be utilized as a potential candidate for evaluation of its G-6-P synthase inhibitory response. Hence, it was planned to synthesize and investigate the naringenin derivatives for their antioxidant, antimicrobial, preservative efficacy and in silico evaluation for G-6-P synthase inhibition.

## Results and discussion

### Chemistry

**Scheme 1:**
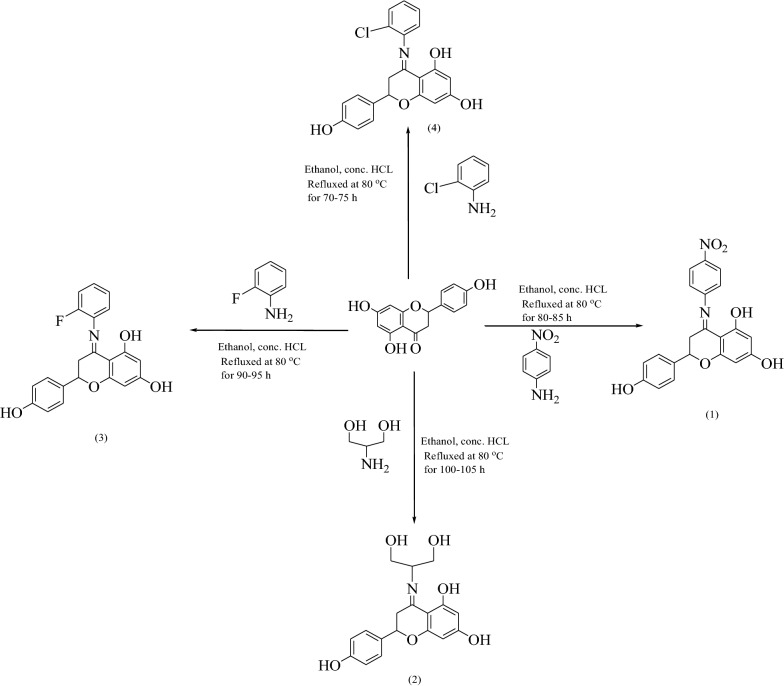
Synthetic route for the synthesis of naringenin derivatives

Naringenin derivatives were synthesized according to Kriza et al. 2011 with slight modifications as shown in Scheme 1 [42]. The chemical structures of all the synthesized compounds were confirmed by FTIR, ^1^H NMR, ^13^C NMR, mass spectroscopy and elemental analysis which were in agreement with the structures.

For the synthesis of naringenin derivatives substituted aniline (0.01 mol) was taken in a round bottom flask and concentrated hydrochloric acid was added drop wise with continuous stirring. Equimolar concentration of naringenin (0.01 mol) was dissolved in ethanol (50 mL) and was refluxed for 80–100 h at 80 °C on heating mantle. All the compounds in series were synthesized according to the standard procedure outlined in Scheme 1. Completion of reaction was confirmed by TLC under UV lamp and FTIR spectra.

Formation of compound **1, 2, 3** and **4** was confirmed by peaks of IR, NMR, mass spectroscopy. In positive chemical ionization most of the naringenin derivatives showed (M++1), M+ (molecular ion peak), (M++2) and in negative chemical ionization mode showed (M+1), (M+2), M+. The elemental analysis established the synthesis of naringenin derivatives where the percentage of C, H and N in the synthesized compounds was observed within defined limits. The reaction mixture was concentrated, after that precipitates formed were filtered off and dried. Crude products were recrystallized by alcohol which yielded the final compounds **1–4**.

### Antioxidant activity

#### DPPH radical scavenging activity

**Fig. 3:**
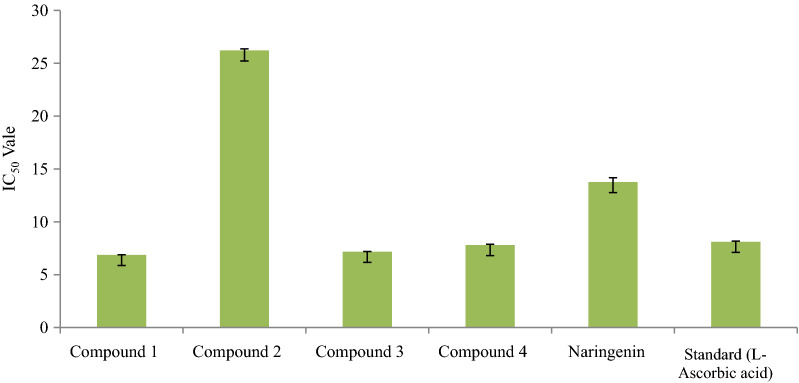
IC_50_ value of different synthesized compunds with respect to standard l-ascarbic acid

All the synthesized compounds were evaluated for antioxidant profile by using DPPH radical scavenging assay method (Table 1). The compound **1** was observed as the most potent antioxidant compound (IC_50_ 6.864 ± 0.020 µM) as compared to standard L-ascorbic acid (IC_50_ 8.110 ± 0.069 µM). However, compounds **3** and **4** showed moderate antioxidant activity (IC_50_ 7.170 ± 0.028 µM and 7.801 ± 0.077 µM, respectively) as compared to standard. The electron withdrawing strongly deactivating nitro group in compound **1** may be responsible for better antioxidant activity. The presence of weakly deactivating electron withdrawing chloro and fluoro groups present in compound 3 and 4 have moderate antioxidant activity. IC_50_ value of synthesized naringenin derivatives has been shown in Fig. 3.

**Table 1:** Antioxidant IC_50_ values of synthesized compounds

S. no.	Compound(s)	IC_50_ (µM)^a^
1.	Compound **1**	*6.864 ± 0.020*
2.	Compound **2**	26.210 ± 0.151
3.	Compound **3**	7.170 ± 0.028
4.	Compound **4**	7.801 ± 0.077
5.	Naringenin	*13.765 ± 0.408*
6.	Standard (l-ascorbic acid)	*8.110 ± 0.069*

^a^Values are expressed as mean ± SEM, n = 3

### Antimicrobial activity

#### Minimum inhibitory concentration

**Fig. 4:**
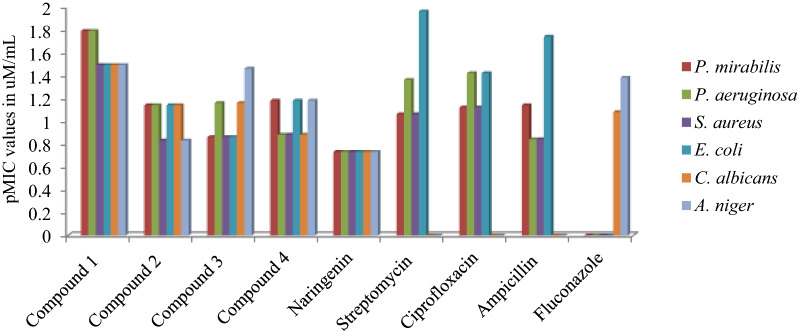
Antimicrobial activity (pMIC in µM/mL) of synthesized naringenin derivatives against different microorganisms

The antimicrobial activity of synthesized compounds revealed compound **1** as the most potent compound (pMIC 1.79, 1.79, 1.49, 1.49, 1.49 and 1.49 μM/mL for *P. mirabilis, P. aeruginosa, S. aureus*, *E. coli, C. albicans* and *A. niger* respectively) as compared to standard drugs taken. The compound **2** showed comparable activity against *P. mirabilis* (pMIC 1.14 μM/mL)*, C. albicans* (pMIC 1.14 μM/mL) while the compound **3** also showed comparable activity against *C. albicans* (pMIC 1.16 μM/mL) as well *A. niger* (pMIC 1.46 μM/mL), likewise the compound **4** showed comparable activity against *P. mirabilis* (pMIC 1.18 μM/mL) as compared to the standard drugs streptomycin (pMIC 1.06, 1.36, 1.06 and 1.96 μM/mL for *P. mirabilis, P. aeruginosa, S. aureus* and *E. coli,* respectively), ciprofloxacin (pMIC 1.12, 1.42, 1.12 and 1.42 μM/mL for *P. mirabilis, P. aeruginosa, S. aureus* and *E. coli,* respectively), ampicillin (pMIC 1.14, 0.84, 0.84 and 1.74 μM/mL for *P. mirabilis, P. aeruginosa, S. aureus* and *E. coli,* respectively) and fluconazole (pMIC 1.08 and 1.38 μM/mL for *C. albicans* and *A. niger,* respectively). In general, the results of MIC studies (Table 2) revealed that the synthesized compounds have better anti bacterial and anti fungal potential as compared to standard drugs streptomycin, ciprofloxacin, ampicillin and fluconazole. The graphically representation of the pMIC values of test and standard compounds have been shown in Fig. 4.

**Table 2:** pMIC values (μM/mL) of synthesized naringenin derivatives against different standard microbial strains

Compound(s)	PMIC values in μM/mL					
	*P. mirabilis*	*P. aeruginosa*	*S. aureus*	*E. coli*	*C. albicans*	*A. niger*
Compound **1**	*1.79*	*1.79*	*1.49*	*1.49*	*1.49*	*1.49*
Compound **2**	1.14	1.14	0.83	1.14	1.14	0.83
Compound **3**	0.86	1.16	0.86	0.86	1.16	1.46
Compound **4**	1.18	0.88	0.88	1.18	0.88	1.18
Naringenin	*< 0.73*	*< 0.73*	*< 0.73*	*< 0.73*	*< 0.73*	*< 0.73*
Streptomycin	*1.06*	*1.36*	*1.06*	*1.96*	–	–
Ciprofloxacin	*1.12*	*1.42*	*1.12*	*1.42*	–	–
Ampicillin	*1.14*	*0.84*	*0.84*	*1.74*	–	–
Fluconazole	–	–	–	–	*1.08*	*1.38*

### Preservative efficacy study

The most active antimicrobial compound **1** was selected for the evaluation of its preservative efficacy. The results of preservative efficacy testing performed in triplicate and were reported as mean values in Table 3.

**Table 3:** Log CFU/mL values of the selected compound **1**

Compound(s)	*E. coli*		*P. aeruginosa*		*S. aureus*		*C. albicans*		*A. niger*	
CFU/mL after days	14 days	28 days	14 days	28 days	14 days	28 days	14 days	28 days	14 days	28 days
Compound **1**	3.190 ± 0.008	3.496 ± 0.12	3.306 ± 0.16	3.406 ± 0.016	3.486 ± 0.012	3.486 ± 0.012	3.200 ± 0.081	3.313 ± 0.016	3.306 ± 0.016	3.463 ± 0.020
Sodium Benzoate	*3.213 ± 0.012*	*3.323 ± 0.24*	*3.282 ± 016*	*3.210 ± 0.037*	*3.863 ± 0.044*	*3.166 ± 0.047*	*3.076 ± 0.088*	*2.800 ± 0.081*	*3.166 ± 0.012*	*3.320 ± 0.014*
Propyl Paraben	*3.280 ± 0.57*	*3.246 ± 0.36*	*3.310 ± 0.016*	*3.306 ± 0.016*	*3.883 ± 0.023*	*3.516 ± 0.012*	*3.940 ± 0.028*	*3.530 ± 0.016*	*3.113 ± 0.065*	*3.403 ± 0.012*
Ethyl Paraben	*3.336 ± 0.020*	*3.090 ± 0.148*	*3.246 ± 0.36*	*3.340 ± 0.014*	*3.166 ± 0.047*	*3.210 ± 0.008*	*3.520 ± 0.014*	*3.200 ± 0.018*	*3.043 ± 0.041*	*3.300 ± 0.081*

# Initial microbial count in inoculums 1 × 10^5^–1 × 10^6^

**Fig. 5:**
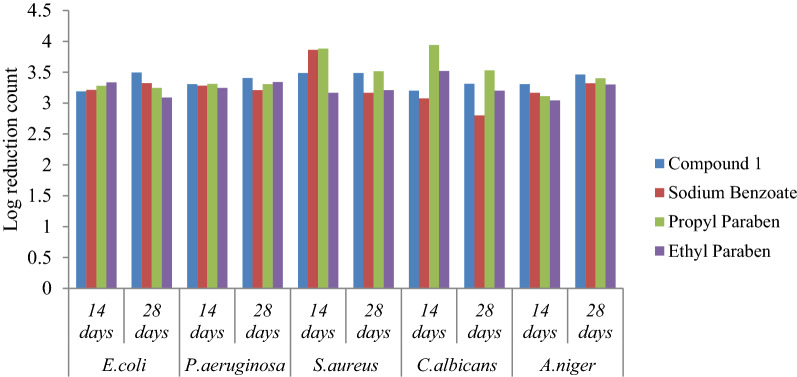
Preservative efficacy of compound **1** in White lotion USP and degree of microbial log reduction

Compound **1** showed the values of log CFU/mL reduction within the prescribed limit and the results were comparable to that of the standard preservatives sodium benzoate, propyl paraben and methyl paraben. The preservative efficacy of compound **1** in White lotion USP and degree of microbial log reduction has been represented in Fig. 5.

### Structure activity relationship (SAR) studies

**Fig. 6:**
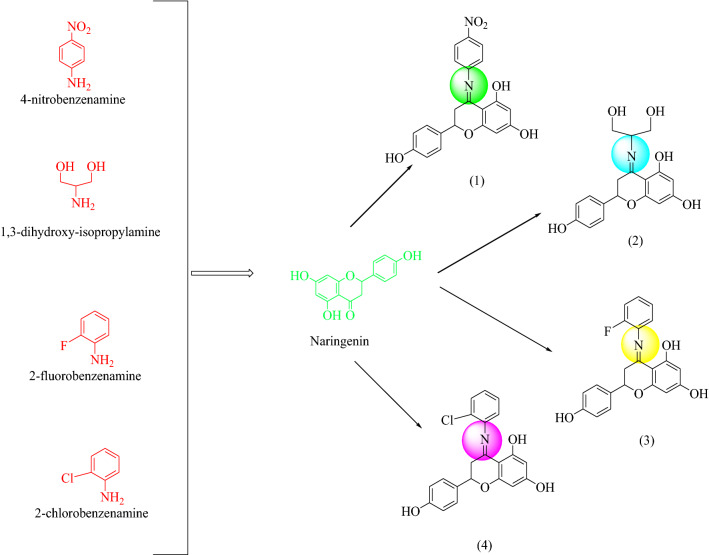
Design strategy of Naringenin derivatives for G-6-P synthase inhibition and antioxidant activity

Design strategy of naringenin derivative for G-6-P inhibition and antioxidant activity has been represented in Fig. 6. The structure activity relationship of the synthesized naringenin derivatives with their antioxidant activity results were summarized as:

1. Substitution of naringenin with aliphatic amines produced biological activity but aromatic substitution showed greater activity than aliphatic i.e. compound **2** showed the lowest activity as compared to other.
2. Substitution with aromatic amine at para position increased the activity with increase in electronegativity i.e. compound **1** was more active than compound **3** and **4**.
3. Replacement of para position with nitro group produced the highest activity i.e. compound **1** was most active in the series.
4. Exchange at para position produced more activity as compared to ortho position substitution.

### Molecular docking study

**Fig. 7:**
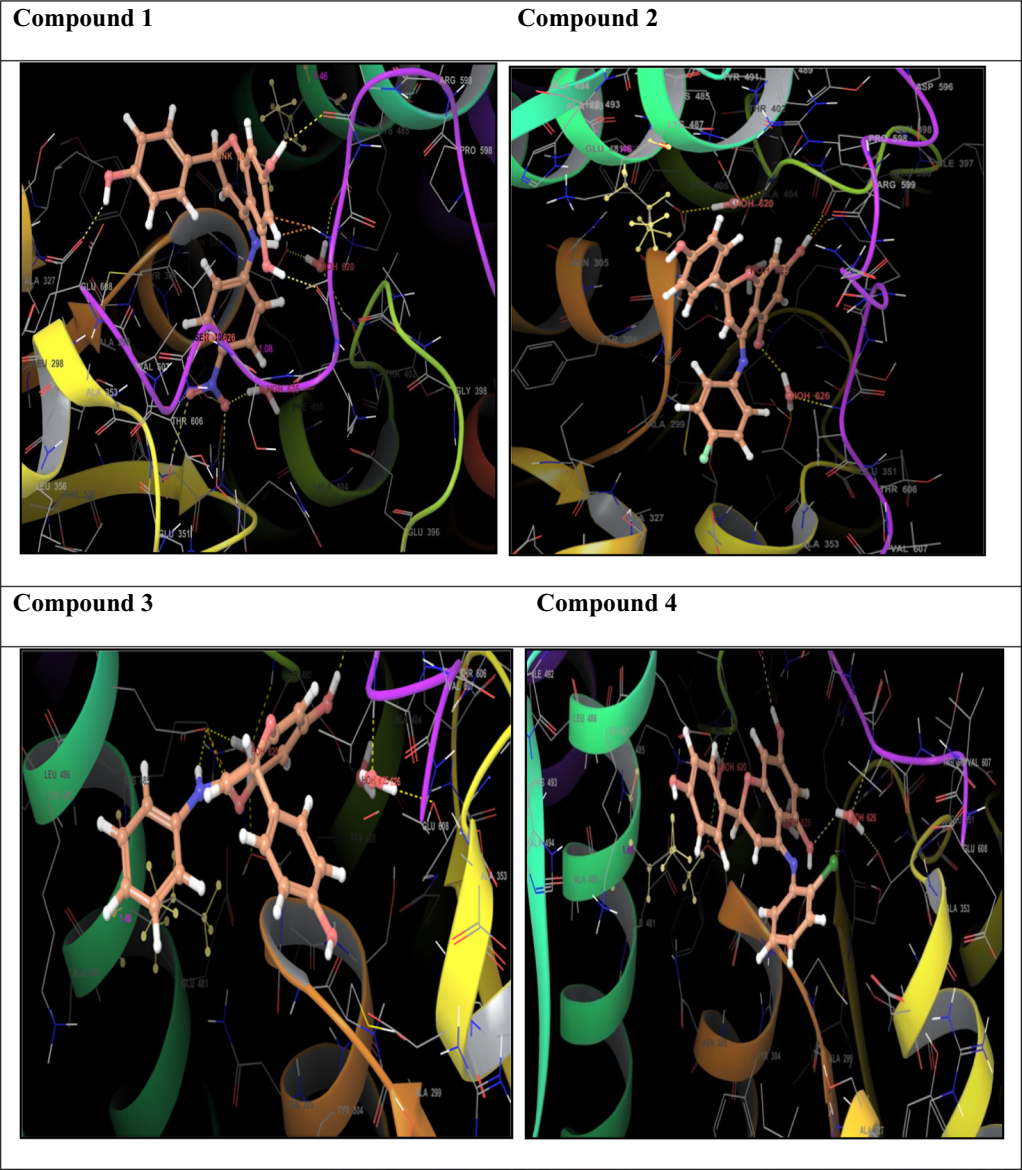
Binding of compounds **1, 2, 3** and **4** with G-6-P synthase

Molecular docking studies were carried out to identify the binding affinities and interaction between the inhibitors and pdb id 1moq of G-6-P synthase protein by using Glide software (Schrodinger Inc. U.S.A. Maestro version 11). Dock score and binding of compound 1, 2, 3 and 4 with G-6-P synthase have been shown in Table 4 and Fig. 7. After, docking results of compound **1** with G-6-P synthase protein suggested the formation of the hydrogen bond between NO_2_ and Thr 402. Additionally, the molecule has been stabilized by residues such as Ser 347, Thr 352, Ser 303, Gln 348, Ala 602, Asn 600 and Asp 354. The binding orientation of compound **2** within the catalytic site of G-6-P synthase exhibited backbone hydrogen bonding with Glu 488. The molecule is stabilized by residues such as Asp 354, Lys 603, Glu 488, Lys 487 and Ala 400. The compound **3** showed interaction with Arg 599. The molecule was enclosed by residues such as Val 399, Thr 302, Lys 487 and Leu 484. In compound **4** hydrogen bonding was shown by Thr 606 and ligand was entrapped by the residue sequence of Val 399, Lys 487, Cys 300 and Ser 328. Docking results of G-6-P synthase showed that the synthetic compounds have comparable docking score as compared to the standard drugs taken. All the ligands showed variable degrees of hydrogen bond interaction, hydrophobic interactions, electrostatic interactions, ionic interactions and π–π stacking with the various amino acid residues in the binding pockets of G-6-P synthase.

**Table 4:** G-6-P synthase inhibition showed by synthesized naringenin derivatives

S. no.	Compound(s)	Structure of G-6-P synthase inhibitors	Dock score
1.	Compound **1**	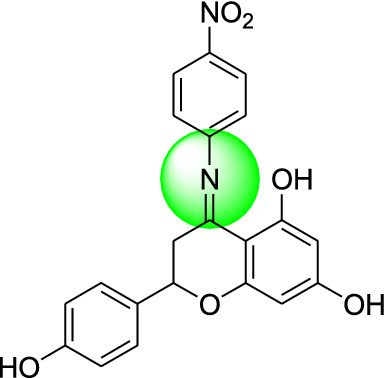	− 7.42
2.	Compound **2**	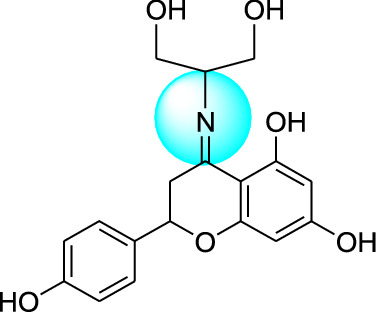	− 4.29
3.	Compound **3**	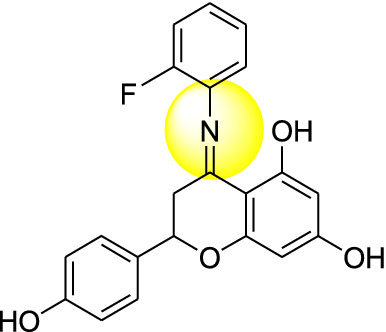	− 3.30
4.	Compound **4**	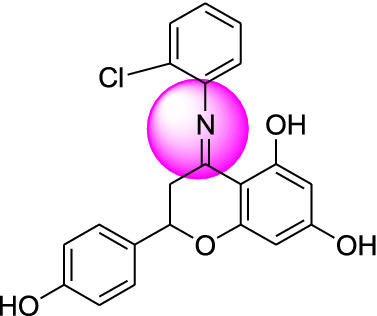	− 4.02
5.	Naringenin	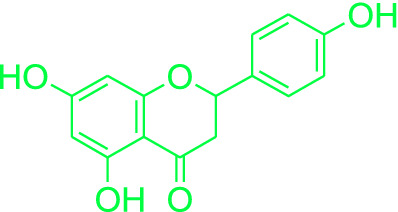	− 6.36
6.	Standard	Streptomycin	*− 5.795*
		Ciprofloxacin	*− 5.185*
		Ampicillin	*− 5.065*
		Fluconazole	*− 5.129*

### ADME study

The evaluation of different ADME parameters has been represented in Table 5. It was observed that all the synthesized compounds fulfilled the standard Rule of Five [43]. All the synthesized compounds qualified the conditions for various descriptors like LogP, HBA, HBD and MW. All these parameters were in suitable range for drug-like characteristics. In addition, according to Veber et al., 2002 for better bioavailability rotatable bonds should be ≤ 10 as the rotatable bonds in ligand impart elasticity [44]. The values of QPlogBB should be > 1.0 CNS active compounds and value < 1.0 CNS inactive compounds. QPPCaco cell permeability should be in a range from 4–70 [45–47]. In the present study, all the synthesized compounds exhibited a suitable drug-like profile.

**Table 5:** ADMET profile of various newly synthesized naringenin derivatives

Compound(s)	Mol. Wt.	No. of rotatable bond	DonorHB	AcceptHB	QPlogPo/w	QPlogBB	QPPMDCK	QPPCaco
Compound **1**	392.10	5	5	4	2.084	0.081	0.053	1.877
Compound **2**	345.12	3	3	3	2.490	0.138	11.251	2.773
Compound **3**	365.11	4	2	4	4.29	2.445	0.282	10.982
Compound **4**	381.08	3	4	2	1.278	3.355	0.162	20.169

## Conclusion

In conclusion, the above mentioned wet and dry laboratory studies highlight the underlying mechanism of G-6-P synthase inhibition. The rational development of inhibitors and specificity of naringenin derivatives to be discovered as the novel preservatives. Moreover, the synthesized compounds were also found as wonderful antioxidants towards DPPH with remarkable potential as compared to the reference compounds.

## Experimental

### Materials and methods

All the chemicals required for experiments were of analytical grade and were purchased from Loba Chemie (Mumbai, India), SRL (Mumbai, India), and Sigma Aldrich (Germany). Nutrient agar, nutrient broth, sabouraud dextrose agar and sabouraud dextrose broth required for antimicrobial and preservative efficacy were obtained from Hi-media Laboratories. Streptomycin, ciprofloxacin, ampicillin and fluconazole were obtained as gift sample from Belco Pharma, Bahadurgarh, India. Microbial strains *S. aureus MTCC* 3160, *P. aeruginosa MTCC* 1934, *E. coli MTCC* 45, *C. albicans MTCC* 183 and *A. niger MTCC* 282 strains were purchased from MTCC, Chandigarh, India. Chemical reactions were monitored by TLC on silica gel plates in iodine and UV chambers. Sonar melting point apparatus in open capillary tube was used for the recording of melting points. ^1^H NMR and ^13^C NMR spectra were confirmed in DMSO and deuterated CDCl_3_ on Bruker Avance II 400 NMR spectrometer at a frequency of 400 MHz downfield to tetramethyl silane standard. FTIR spectra were recorded on Perkin Elmer FTIR spectrophotometer with the help of KBr pellets technique. Waters Micromass Q-ToF Micro instrument was used for Mass spectrum recording.

### General procedure for the synthesis of naringenin derivatives

Substituted aniline (0.01 mol) was taken in a round bottom flask, concentrated hydrochloric acid was added drop wise with continuous stirring. Equimolar concentration of naringenin (0.01 mol) was dissolved in ethanol (50 mL) and was re fluxed for 80-100 h on heating mantle. All the compounds in the series were synthesized according to the standard procedures as outlined in Scheme 1. Completion of reaction was monitored by TLC. Reaction mixture was concentrated and the precipitates formed were filtered off and dried. The crude product was recrystallized using alcohol which yielded the final compounds **1-4**.

### Spectral data

#### 2-(4-hydroxyphenyl)-4-(4-nitrophenylimino) chroman-5, 7-diol

*R*_*f*_ TLC mobile phase: Chloroform: Acetone (8:5) = 0.63; Yield = 55%; M.P. = 190–192 °C; M.Wt. = 317.29; IR (KBr pellets) cm^−1^: 1081 (–C–O–C), 1156 (–C–C–), 1305 (–NO_2_), 1599 (–C=C–), 1632 (–C=N–), 2921 (–C–H–), 3479 (–OH–); ^1^H NMR (400 MHz, DMSO-*d*_6_) δ = 11.94 (s, 1H), 11.10 (s, 1H), 8.17 (d, *J* = 8.5 Hz, 2H), 8.04 (s, 1H), 7.29 (d, *J* = 9.3 Hz, 2H), 7.28 (d, *J* = 7.5 Hz, 2H), 6.80 (d, *J* = 7.3 Hz, 2H), 6.28 (s, 1H), 6.27 (s, 1H), 5.28 (t, *J* = 9.0 Hz, 1H), 3.15 (d, *J* = 7.3 Hz, 1H), 3.00 (d, *J* = 7.3 Hz, 1H); ^13^C NMR (400 MHz, CDCL_3_) δ = 166.11, 165.34, 163.98, 161.90, 153.71, 152.59, 146.42, 133.96, 131.14, 126.43, 125.72, 124.08, 123.64, 117.42, 103.05, 97.89, 95.36, 77.13, 38.79, 27.19, 22.70; MS ES + (ToF): m/z 392.10 [M^+^+2]; CHNS: Calc (C_12_H_16_N_2_O_2_): C, 64.28; H, 4.11; N, 7.14; O, 24.47; Found C, 64.25; H, 4.14; N, 7.17; O, 24.44.

#### 4-(1,3-dihydroxypropan-2-ylimino)-2-(4-hydroxyphenyl)chroman-5,7-diol

*R*_*f*_ TLC mobile phase: Chloroform: Acetone (8:5) = 0.66; Yield = 50%; M.P. = 173–175 °C; M.Wt. = 345.32; IR (KBr pellets) cm^−1^: 1074 (–C–O–C–), 1251 (–C–C–), 1513 (–C=C–), 1631 (–C=N–), 2831 (–C–H–), 3295 (–OH–); ^1^H NMR (400 MHz, DMSO-*d*_6_) δ = 11.60 (s, 1H), 11.10 (s, 1H), 8.04 (s, 1H), 7.28 (d, *J* = 7.2 Hz, 2H), 6.80 (d, *J* = 7.3 Hz, 2H), 6.28 (s, 1H), 6.24 (s, 1H), 5.25–5.24 (m, 1H), 3.95 (d, *J* = 8.1 Hz, 2H), 3.64 (q, *J* = 9.0 Hz, 2H), 3.50 (q, *J* = 9.6 Hz, 2H), 3.47–3.45 (m, 1H), 3.13 (d, *J* = 8.6 Hz, 1H), 2.86 (d, *J* = 8.6 Hz, 1H); ^13^C NMR (400 MHz, CDCL_3_) δ = 164.81, 162.47, 161.87, 161.58, 158.28, 130.84, 128.42, 115.98, 107.15, 103.33, 96.95, 78.20, 72.30, 63.75, 37.92, 27.60, 22.32, 14.16; MS ES + (ToF): m/z 345.12 [M^+^+2]; CHNS: Calc (C_18_H_19_NO_6_): C, 62.60; H, 5.55; N, 4.06; O, 27.80; Found C, 62.63; H, 5.52; N, 4.09; O, 27.82.

#### 4-(2-fluorophenylimino)-2-(4-hydroxyphenyl)chroman-5,7-diol

*R*_*f*_ TLC mobile phase: Chloroform: Acetone (8:5) = 0.64; Yield = 23%; M.P. = 165-167 °C; M.Wt. = 365.35; IR (KBr pellets) cm^−1^: 753 (–F–), 1082 (–C–O–C), 1241 (–C–C–), 1612 (–C=C–), 1632 (–C=N–), 2833 (–C–H–), 3350 (–OH–); ^1^H NMR (400 MHz, DMSO-*d*_6_) δ = 11.78 (s, 1H), 11.10 (s, 1H), 8.04 (s, 1H), 7.47 (d, *J* = 8.8 Hz, 1H), 7.31 (dt, *J* = 15.7, 8.4 Hz, 2H), 7.28–7.26 (m, 3H), 6.80 (d, *J* = 7.4 Hz, 2H), 6.31 (s, 1H), 6.28 (s, 1H), 5.33 (t, *J* = 8.5 Hz, 1H), 3.04 (d, *J* = 7.7 Hz, 1H), 2.92 (d, *J* = 8.5 Hz, 1H); ^13^C NMR (400 MHz, CDCL_3_) δ = 165.92, 165.91, 165.24, 163.73, 161.86, 132.64, 132.61, 126.96, 126.94, 126.50, 126.48, 125.25, 114.89, 114.86, 102.91, 97.83, 95.53, 72.64, 39.18, 20.46; MS ES+ (ToF): m/z 365.11 [M^+^+2]; CHNS: Calc (C_21_H_16_FNO_4_): C, 69.04; H, 4.41; F, 5.20; N, 3.83; O, 17.52; Found C, 69.01; H, 4.44; F, 5.23; N, 3.84; O, 17.55.

#### 4-(2-chlorophenylimino)-2-(4-hydroxyphenyl)chroman-5,7-diol

*R*_*f*_ TLC mobile phase: Chloroform: Acetone (8:5) = 0.66; Yield = 60%; M.P. = 155-157 °C; M.Wt. = 381.81; IR (KBr pellets) cm^−1^: 754 (–Cl–Str), 1062 (–C–O–), 1155 (–C–C–), 1602 (–C=C–) 1633 (–C=N–), 2834 (–C–H–), 3284 (–OH–); ^1^H NMR (400 MHz, DMSO-*d*_6_) δ = 11.78 (s, 1H), 11.10 (s, 1H), 8.04 (s, 1H), 7.55 (d, *J* = 6.9 Hz, 1H), 7.39 (t, *J* = 8.0 Hz, 1H), 7.28 (d, *J* = 8.0 Hz, 2H), 7.26 (d, *J* = 8.3 Hz, 1H), 7.17 (d, *J* = 7.6 Hz, 1H), 6.80 (d, *J* = 7.5 Hz, 2H), 6.19 (s, 1H), 6.17 (s, 1H), 5.34 (t, *J* = 8.9 Hz, 1H), 3.04 (d, *J* = 8.7 Hz, 1H), 2.94 (d, *J* = 9.1 Hz, 1H); ^13^C NMR (400 MHz, CDCL_3_) δ = 165.10, 163.08, 161.26, 159.81, 143.28, 139.86, 129.24, 128.98, 128.45, 128.28, 127.73, 127.42, 126.85, 124.29, 107.38, 102.08, 95.02, 76.72, 38.77, 17.39, 14.71; MS ES+ (ToF): m/z 381.08 [M^+^+2]; CHNS: Calc (C_21_H_16_ClNO_4_): C, 66.06; H, 4.22; Cl, 9.29; N, 3.67; O, 16.76; Found C, C, 66.09; H, 4.20; Cl, 9.26; N, 3.69; O, 16.72.

### Antioxidant activity

#### DPPH radical scavenging assay

Antioxidant activity of the synthesized compounds was determined by DPPH (2, 2-diphenyl-1-pycrilhydrazil hydrate) radical scavenging method. Briefly, 0.1 mM solution of DPPH in methyl alcohol was prepared and 1 mL of this solution was added to 3 mL of sample or standard with a concentration of 12.5, 25, 50, 75 and 100 μg/mL. Discolorations were measured at 517 nm after incubation for 30 min at 30 °C in the dark. Lower absorbance of the reaction mixture indicates higher free radical scavenging activity. The IC_50_ values of given samples were calculated by using formula:

$$ {\text{IC}}_{ 50} \, = \,\left( {{\text{A}}_{\text{c}} - {\text{A}}_{\text{s}} } \right)\, \times \, 100/{\text{A}}_{\text{c}} $$

Here, A_c_ was the absorbance of the control and A_s_ was the absorbance of the sample [48, 49].

### Antimicrobial activity

#### Minimum inhibitory concentration (MIC)

The antimicrobial activity of the synthesized compounds were performed against *S. aureus MTCC* 3160, *P. aeruginosa MTCC* 1934, *E. coli MTCC* 45, *P. mirabilis MTCC* 3310*, C. albicans MTCC* 183 and *A. niger MTCC* 282 by using the tube dilution method [50]. Dilutions of test and standard compounds were prepared in double strength nutrient broth I.P. (bacteria) or sabouraud dextrose broth I.P. (fungi) [51, 52]. The slants of *E. coli*, *P. aeruginosa, P. mirabilis* and *S. aureus* were incubated at the 30-35 °C for 24 h. The slants of *C. albicans* were incubated at 20–25 °C for 48 h whereas; the slants of *A. niger* were incubated at 20–25 °C for 5 days. After the incubation period sterilized 0.9% NaCl solution was used to harvest the bacterial and fungal cultures from agar slant through proper shaking and then the suspensions of microorganisms were diluted with the sterile 0.9% NaCl solution to CFU count was adjusted by adjusting the density of microorganism suspension to that of 0.5 McFarland standards by adding distilled water. The number of CFU was determined by dilution pour-plate method [53]. A serial dilution of 50 µg/mL, 25 µg/mL, 12.5 µg/mL, 6.25 µg/mL, 3.12 µg/mL and 1.62 µg/mL was used for determination of MIC. The samples tubes were incubated at 37 °C for 24 h (bacteria), at 25 °C for 7 days (*A. niger*), and at 37 °C for 48 h (*C. albicans*) and the results were recorded in pMIC.

#### Preservative effectiveness

White lotion USP was utilized as the medium for the testing of preservative effectiveness.

Ingredients: Zinc sulfate 40 gm, sulfurated potash 40 gm and purified water q.s. to 1000 mL.

Firstly, zinc sulphate and sulfurated potash were dissolved in 450 mL of water separately and filtered. Then, sulfurated potash solution was added to zinc sulfate with stirring. At last, the required amount of water was added and mixed thoroughly and sterilized. For preservative efficacy testing, the White lotion USP was prepared using the equimolar amount of compounds **1-4** as novel preservatives by replacing sodium benzoate, methyl paraben and propyl paraben from the formula [54].

#### Challenge microorganism

*Staphylococcus aureus MTCC* 3160, *P. aeruginosa MTCC* 1934, *E. coli MTCC* 45, *C. albicans MTCC* 183 and *A. niger MTCC* 282 were used as common contaminants in the study as prescribed in USP for preservative efficacy testing in the pharmaceutical preparations.

#### Preparation of ioculums

The slants of *E. coli*, *P. aeruginosa* and *S. aureus* were incubated at the 30–35 °C for 24 h. The slants of *C. albicans* were incubated at 20–25 °C for 48 h whereas; the slants of *A. niger* were incubated at 20–25 °C for 5 days [55].

#### Test procedure

White lotions USP was added in final containers and were used in challenge test. The preparation was inoculated with 0.5–1% volume of microbial inoculum having a concentration of 1 × 10^5^–1 × 10^6^ CFU/mL [56]. Inoculated samples were mixed thoroughly to ensure homogeneous microorganism distribution and incubated. The CFU/mL of the product was determined at an interval of 0 days, 7 days, 14 days, 21 days, and 28 days in agar plates. Log CFU/mL of white lotion USP was calculated as not less than 2.0 log reductions from initial count at 14 days of incubation and no increase in CFU from 14 days count at 28 days in case of bacteria and no increase from the initial calculated count at 14 and 28 days [57].

#### In silico molecular docking studies

The Schrodinger, Inc. (New York, USA) software Maestro 11 was used for the computational calculations and docking studies. Laboratory for Enzyme Inhibition Studies, Department of Pharmaceutical Sciences, M.D. University, Rohtak, INDIA was used for the computational work. The receptor-grid files were generated by grid-receptor generation program Glide [58]. Grid-based ligand docking utilized the hierarchical sequence of filters to produce possible conformations of the ligand in the active-site region of the protein receptor. At this stage, crude score values and geometric filters were prepared out unlikely binding modes. The next filter phase involves a grid-based force field evaluation and refinement of docking experiments including torsional and rigid-body movements of the ligand [59]. The remained docking evaluations were subjected to a Monte Carlo procedure to minimize the energy score. A conjugate gradient minimization protocol was used in all calculations [60].

The energy differences were calculated using the equation:

$$ \Delta E\, = \,E_{complex} \, - \,E_{ligand} \, - \,E_{protein} $$

### Protein preparation

The X-ray protein structure co-ordinates of pdb id 1moq were downloaded from Protein Data Bank from www.rcbs.org [61] and were prepared with the help of the Schrödinger protein preparation wizard ‘Prepwiz’ [62, 63]. PDB id 1moq (resolution 1.57 A°) was selected on the basis of the lowest resolution and availability. All the waters molecules except metals co-ordinated and present between the ligand and protein were removed. The energy-restrained structure of the protein G-6-P synthase was constructed with the help of OPLS-2005 force field.

### Ligand Preparation

The three-dimensional structural library was prepared using the Chemdraw software and proceeded for energy minimization using the LigPrep tool for the correction of coordinates, ionization, stereochemistry and tautomeric structure to gain the appropriate conformation through the addition or removal of hydrogen bonds. The partial charges were computed according to the OPLS-2005 force field (32 stereo isomers, tautomers and ionization) at biological pH and used for molecular docking studies.

## Data Availability

The datasets used and/or analysed during the current study are available from the corresponding author on reasonable request.

## Abbreviations

ADMET: Absorption, distribution, metabolism, excretion & toxicity
G-6-P Synthase: Glucosamine-6-phosphate synthase
CYP P450: Cytochromes P450
OATP1B1: Solute carrier organic anion transporter family member 1B1
DHEAS: Dehydroepiandrosterone
PPAR: Peroxisome proliferator-activated receptors
DPPH: 2,2-Diphenyl-1-picrylhydrazyl
UDP-N-acetyl glucosamine: Uridine diphosphate N-acetylglucosamine
FTIR: Fourier-transform infrared spectroscopy
^1^H NMR: Proton nuclear magnetic resonance
^13^C NMR: Carbon 13 nuclear magnetic resonance
UV: Ultra violet
TLC: Thin layer chromatography
IC_50_: Inhibitory concentration
MIC: Minimum inhibitory concentrations
CFU: Colony forming unit
HBA: Hydrogen bond acceptor
HBD: Hydrogen bond donor
MW: Molecular weight
MTCC: Microbial type culture collection
DMSO: Dimethyl sulfoxide
BOD: Biological oxygen demand
USP: United States Pharmacopoeia
PDB ID: Protein Data Bank Identification
OPLS: Optimized potential for liquid simulations
Q.S.: Quantity sufficient
